# Bacterial Monitoring with Adhesive Sheet in the International Space Station-“Kibo”, the Japanese Experiment Module

**DOI:** 10.1264/jsme2.ME12184

**Published:** 2013-04-20

**Authors:** Tomoaki Ichijo, Hatsuki Hieda, Rie Ishihara, Nobuyasu Yamaguchi, Masao Nasu

**Affiliations:** 1 Graduate School of Pharmaceutical Sciences, Osaka University, 1–6 Yamadaoka, Suita, Osaka 565–0871, Japan; 2 School of Pharmaceutical Sciences, Osaka University, 1–6 Yamadaoka, Suita, Osaka 565–0871, Japan

**Keywords:** International Space Station (ISS), bacterial monitoring, adhesive sheet, Japanese Experiment Module “Kibo”

## Abstract

Microbiological monitoring is important to assure microbiological safety, especially in long-duration space habitation. We have been continuously monitoring the abundance and diversity of bacteria in the International Space Station (ISS)-“Kibo” module to accumulate knowledge on microbes in the ISS. In this study, we used a new sampling device, a microbe-collecting adhesive sheet developed in our laboratory. This adhesive sheet has high operability, needs no water for sampling, and is easy to transport and store. We first validated the adhesive sheet as a sampling device to be used in a space habitat with regard to the stability of the bacterial number on the sheet during prolonged storage of up to 12 months. Bacterial abundance on the surfaces in Kibo was then determined and was lower than on the surfaces in our laboratory (10^5^ cells [cm^2^]^−1^), except for the return air grill, and the bacteria detected in Kibo were human skin microflora. From these aspects of microbial abundance and their phylogenetic affiliation, we concluded that Kibo has been microbiologically well maintained; however, microbial abundance may increase with the prolonged stay of astronauts. To ensure crew safety and understand bacterial dynamics in space habitation environments, continuous bacterial monitoring in Kibo is required.

The International Space Station (ISS) has been staffed continuously since the first resident crew entered the facility on 2 November 2000, thereby providing a permanent human presence in space. During space flight, a variety of physiological and psychological stressors associated with the space environment, and spacecraft conditions, potentially contribute to detrimental alterations in the human immune system (1). In addition, microorganisms that might present a health hazard to the crew and potentially damage flight hardware have been recognized in the ISS (3, 13, 14). Space agencies have attempted to avoid microbiological problems by developing strategies to limit contamination aboard the ISS (11) (Japan Aerospace Exploration Agency [JAXA] website, ISS/Kibo utilization scenario toward 2020, http://iss.jaxa.jp/kiboexp/news/2020_kibo.html [in Japanese];Towards Human Exploration of Space: a European Strategy [THESEUS] website, THESEUS Roadmap, http://theseus.hd20.hosting.punkt.de/fileadmin/Docs/Eg_reports_roadmap/RoadMap_web.pdf).

For microbial monitoring of interior surfaces of the ISS, sampling has been performed with swabs or agar-based media (10, 13), and laboratory analysis of bacterial genera is carried out back on Earth. These conventional sampling methods have some problems that should be resolved: use of water for swabbing methods; potential risk of injury if the handle of the swab is broken during sampling; contamination of surfaces with culture medium components; and difficulty of sampling from curved surfaces. For sampling microbial cells on solid surfaces, we have developed a microbe-collecting adhesive sheet (20). This sheet has high operability, needs no water for sampling, and is easy to transport and store. In the present study, we evaluated via a laboratory test on ground the applicability of the adhesive sheet for microbial monitoring in a space habitat by determining the effect of preservation of the adhesive sheet on the collected microbes, because sample storage is necessary before transporting back to the laboratory on Earth.

We then used the adhesive sheet for microbial monitoring in the ISS. The environment in the ISS is different from that on Earth, and the microbial ecosystem may also differ. Therefore, investigation is required of the relationship between humans and microbes, as well as how microbes influence the materials and systems in this closed environment. We have been continuously monitoring microbes in the Japanese Experiment Module “Kibo” on the ISS, in cooperation with the Japan Aerospace Exploration Agency (JAXA) (research title: “Microbe”: http://iss.jaxa.jp/en/kiboexp/news/101101_microbe-2_start.html). The objective of “Microbe” is to monitor changes in microbial abundance and species diversity in Kibo, which entered operation in June 2008, and to clarify microbial dynamics in space habitation systems. In 2010, microbes were collected by applying the adhesive sheets to the surfaces of the interior and the equipment in Kibo. After the samples were transported to our laboratory, bacterial abundance and taxonomic distribution were determined.

## Materials and Methods

### Bacterial strains

The following bacterial species have been detected in the ISS, and reference strains of these species were used in this study: *Acinetobacter lwoffii* ATCC15390, *Bacillus subtilis* 168, *Pseudomonas putida* ATCC12633 and *Staphylococcus epidermidis* IFO3762. Each bacterial strain was incubated in LB broth at 37°C until reaching the stationary phase.

### Sample preparation for evaluating recovery rate and preservation experiment

A painted stainless plate was provided by JAXA (Tokyo, Japan) for the following ground experiments. The plate was disinfected by spraying 70% ethanol on all surfaces and wiping with a paper towel immediately before performing each experiment.

**Fig. 1:**
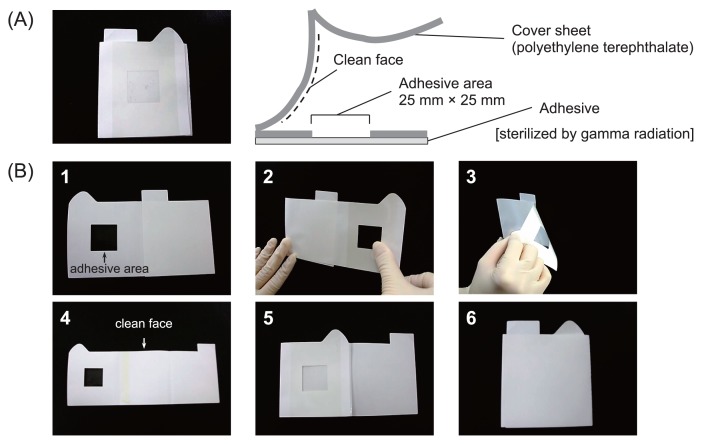
Adhesive sheet for microbial monitoring in space habitat. Sheets were sterilized by gamma radiation and packed individually in small bags. (A) Photograph and schematic illustration of adhesive sheet. (B) Procedure for microbial sampling: 1, open triangular tab; 2, attach adhesive area to sampling site and press three times; 3, peel adhesive sheet off sampling site; 4, open rectangular tab; 5, close triangular tab; 6, close rectangular tab.

To evaluate the recovery rate, 1.0×10^7^ mixed cultured cells (2.5×10^6^ cultured cells each) suspended in sterilized water were spread on a 6.25 cm^2^ area on the plate. After the cells were air-dried, they were recovered with an adhesive sheet (Fig. 1) or a swab (Large Alpha Swab; TexWipe, Kernersville, NC, USA). To achieve a high recovery rate of bacterial cells with the adhesive sheet, the adhesive face was pressed down three times (repeatedly placing and removing the same sheet) in exactly the same area (20). Swabbing was optimized and performed as described previously (18). Recovered cells were fluorescently stained with 1×SYBR Green II (Invitrogen, Carlsbad, CA, USA) and counted under an epifluorescent microscope (Eclipse E400; Nikon, Tokyo, Japan) at a magnification of ×1,000. Recovery rate was calculated by dividing the number of recovered bacterial cell by the spread cell number (1.0×10^7^).

For the preservation experiment, 1.8×10^5^ mixed cultured cells (4.5×10^4^ cultured cells each) suspended in sterilized water were spread on a 6.25 cm^2^ area on the plate and recovered with the adhesive sheet. Sampling with the adhesive sheet was performed as described above. The adhesive sheets on which bacterial cells were collected were packed in small Ziplock bags and stored at −80°C, 4°C or room temperature (approx. 20°C) for one to 12 months. Samples without any preservation were used as the time zero control. Bacterial cells on the adhesive sheet were fluorescently stained with 1×SYBR Green II after storage. Recovered cells in 20 microscopic fields were counted under the epifluorescent microscope at ×1000 magnification. Recovery rate was calculated by dividing the number of fluorescently stained cells after storage by that on the initial day.

### Sampling in Kibo

In Kibo, sampling using adhesive sheets was performed by an astronaut on 29 October 2010 at 1830 JST (880 d after launch) as part of the “Microbe” research program. The surfaces of the CBEF (Cell Biology Experiment Facilities; relatively low frequency of astronaut contact), PC palm rest (routine contact by astronauts), return air grill (intake of air) and handrail (high frequency of astronaut contact) were selected as sampling sites. Samples were stored in the freezer (−80°C) installed in Kibo for seven months and transferred to our laboratory via NASA Kennedy Space Center and JAXA Tsukuba Space Center at −80°C. We received the samples on 6 June 2011 (JST).

### Total direct counting of microbes collected with adhesive sheet

Center of the adhesive area (5 mm×5 mm) was excised aseptically with a sterilized razor. This piece of sheet was used for total direct counting of microbes. The other area was used for DNA extraction as described below. Collected cells on the adhesive sheet were fluorescently stained with 1×SYBR Green II. Fluorescently stained cells in 50 microscopic fields were counted under the epifluorescent microscope at ×1,000 magnification.

### DNA extraction from adhesive sheet sample

The adhesive area used for DNA extraction (6 cm^2^) was excised aseptically with scissors. DNA was extracted with the Fast DNA Spin Kit for Soil (MP Biomedicals, Irvine, CA, USA). The excised sheet for DNA extraction was inserted into a Lysing Matrix E tube provided as part of the kit. After insertion, 2 μL luciferase gene fragment was added as an internal control for quantitative PCR (12), and then DNA extraction was performed following the manufacturer’s instructions. For dilution of DNA, 100 μL DES was used, provided as part of the kit.

### Quantitative PCR

For determination of bacterial abundance, the 16S rRNA gene was quantified by quantitative PCR with a Light Cycler (Roche Diagnostics, Mannheim, Germany) (19). Quantitative PCR amplification was performed with the reagents supplied with the Light Cycler DNA Master SYBR Green I (Roche Diagnostics). The quantitative PCR mixture, containing 4 mM MgCl_2_, 0.5 μM each primer (EUB f933, EUB r1387; Table 1) and 4.5 ng μL^−1^ 8-methoxypsoralen was made up to 8 μL with DNA-free water. The 10× Light Cycler DNA Master SYBR Green I and the DNA suspension were added last in 1 μL volume each after irradiation of the PCR mixture with UV light (7). After an initial denaturing step at 95°C for 10 min, 45 cycles were performed as follows: denaturing at 95°C for 15 sec, annealing at 60°C for 10 sec, extension at 72°C for 30 sec, and signal detection at 86°C for 5 sec.

To determine the rate of recovery of DNA during extraction, known amounts of PCR products of the luciferase gene (*luc*) were inoculated into the samples as an internal standard and quantified after DNA extraction according to Nishimura *et al.* (12). The DNA recovery rate was calculated by comparing the copy number of the inoculated *luc* gene before and after DNA extraction. The copy number of the 16S rRNA gene quantified by quantitative PCR was calibrated based on the recovery rate.

### Nested PCR-DGGE analysis

16S rRNA gene fragments were amplified using the primers EUB f933-GC-clamp and EUB r1387 following amplification of nearly the full-length 16S rRNA gene with 8f and 1492r primer set (Table 1). First and second PCR amplifications were performed as described by Buchholz-Cleven *et al.* (2) and Iwamoto *et al.* (6), respectively. Denaturant gradient gel electrophoresis (DGGE) was run as described by Iwamoto *et al.* (6). Briefly, 200 ng PCR product was loaded onto a 6.5% (w/v) polyacrylamide gel cast in 1×TAE (40 mM Tris, 20 mM acetic acid, and 1 mM EDTA; pH 8.0). Polyacrylamide gels (acrylamide : bisacrylamide, 37.5:1) were made with denaturing gradients ranging from 45 to 65%. After electrophoresis, DGGE gel was soaked in 1×SYBR Gold (Invitrogen) for 30 min.

The bands in the DGGE gel were excised with a sterilized razor blade under blue excitation light, and then placed in 100 μL nucleic acid-free water. After overnight incubation at 4°C, the supernatant was used as a template for re-amplification with EUB f933 and EUB r1387 primer set (Table 1). Because a single DGGE band would not represent a single bacterial strain (17), we constructed small clone libraries using the pGEM-T Easy Vector System II (Promega, Madison, WI, USA) and inserted fragments of randomly selected six clones were sequenced (Hokkaido System Sciences, Hokkaido, Japan). Nucleic acid sequences were analyzed by the ribosomal database project (4) in order to determine the taxonomic distribution. A phylogenetic tree was constructed by the neighbor-joining method.

### Nucleotide sequence accession numbers

The sequences obtained from the DNA clone library were deposited in the DNA Data Bank of Japan (DDBJ) database under accession numbers AB720834 to AB720837 (CBEF surface), AB720838 to AB720850 (handrail), AB720851 to AB720853 (return air grill) and AB720854 to AB720871 (PC palm rest).

### Statistical analysis

Student’s t test was carried out using an online statistical analysis program MEPHAS (http://www.gen-info.osaka-u.ac.jp/testdocs/tomocom/).

## Results and Discussion

Sampling is one of the most important processes in environmental microbiology. In this study, we first evaluated the applicability of our adhesive sheet for bacterial monitoring in a space habitat. Sampling in the ISS is not performed by specialists in microbiology but by those who are not familiar with this field, such as astronauts; thus, any sampling technique should have high operability. The microbe-collecting adhesive sheet developed for the present study is shown in Fig. 1A. This sheet was 7 cm×8.5 cm (adhesive area: 2.5 cm×2.5 cm). To avoid any microbial contamination of the clean face, the cover sheet is folded in half. Sheets were packed individually in a small bag and sterilized by gamma radiation (10 kGy; Koga Isotope, Shiga, Japan). The sampling procedure is shown in Fig. 1B. Before sampling, we confirmed by fluorescent staining that there were no microbial cells on the adhesive area. In order to protect the adhesive area of the sheet from any contamination, the clean face (inside of the cover sheet) is covered during sampling, as shown in Fig. 1B (step 1–3). After sampling, the adhesive area faces the clean face (Fig. 1B, step 4, 5), and is stored (Fig. 1B, step 6).

**Fig. 2:**
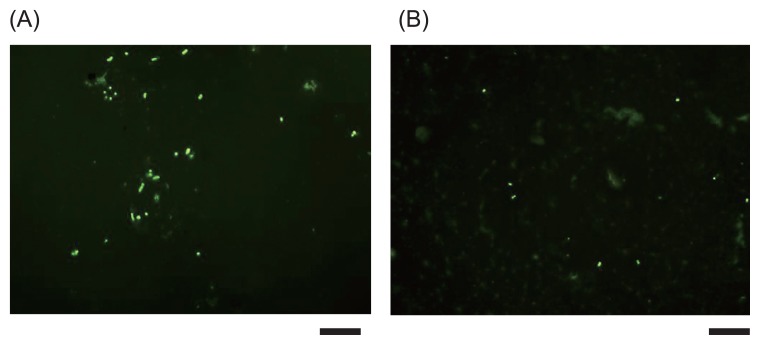
Fluorescent microscopic image of microbes collected with an adhesive sheet. Microbial cells were stained with 1×SYBR Green II (scale bars, 10 μm). (A) Mixture of *A. lwoffii* ATCC15390, *B. subtilis* 168, *P. putida* ATCC12633 and *S. epidermidis* IFO3762; (B) sample from vertical surface of the rack in our laboratory.

Microbial cells collected on adhesive sheets were directly stained with 1×SYBR Green II (Fig. 2). Four mixed species of cultured bacteria and microbes collected from the vertical surface of the rack in our laboratory (ground control of CBEF surface in Kibo) were used as samples. As shown in Fig. 2, microbial cells stained with SYBR Green II were clearly observed with an epifluorescent microscope.

Next, we evaluated the recovery rate of bacterial cells spread on the painted stainless plate with the adhesive sheet. A swab was also used for sampling and the results were compared. The recovery rates of bacterial cells with the adhesive sheet and the swab were 78±12% (*n*=5) and 69±11% (*n*=10), respectively. The ability of the adhesive sheet to collect microbes from a solid surface was equivalent to that of the swab (*P*>0.05; Student’s t test). We also evaluated the recovery rate of bacterial cells spread on the laptop palm rest (plastic, rough surface) with the adhesive sheet. The rate was calculated as 71±6.1% (*n*=3). Using the adhesive sheet, the recovery rate from the plastic surface was not significantly different from that from the painted stainless plate (*P*>0.05; Student’s t test). The adhesive sheet was therefore an alternative device for sampling in a space habitat.

**Fig. 3:**
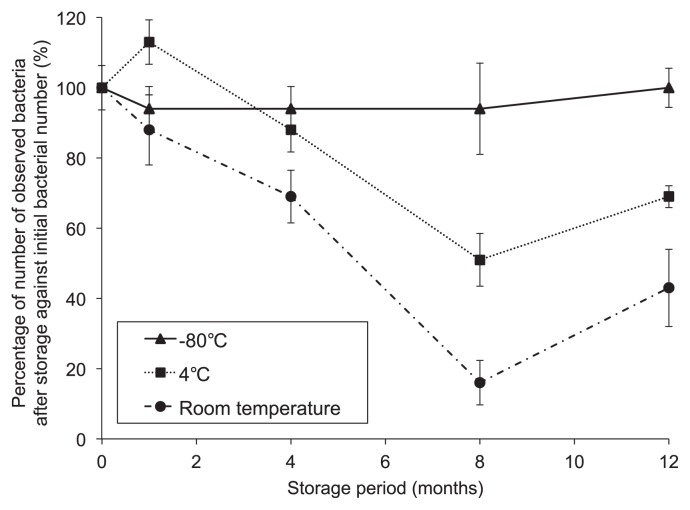
Changes in numbers of recovered bacterial cells on adhesive sheets during prolonged storage. Initial bacterial number was converted to 100%. Error bars show standard deviations (*n*=3).

For bacterial monitoring in the ISS, it is difficult to analyze the samples immediately because transportation to the ground laboratory strictly depends on the availability of the return vehicle. In some cases, long-term storage in the ISS will be required. Changes in bacterial number during prolonged storage at various temperatures were therefore determined (Fig. 3). The number of SYBR Green II-stained bacterial cells that could be counted by microscopy on adhesive sheets stored at room temperature and 4°C markedly decreased during storage. In contrast, the number of bacteria collected on the adhesive sheet and stored at −80°C was >90% of the initial number and did not significantly change during this period (until 12 months) (*P*<0.05; Student’s *t* test). We also evaluated the effect of long-term storage at −80°C for eight months on the number of 16S rRNA genes, which was stable for one to eight months, as was the bacterial number determined by fluorescent microscopy. Therefore, after storing samples at −80°C, the counts obtained reflect the abundance of bacteria collected on the day of sampling (time zero). The sampling sheet enables us to analyze the bacteria in the ISS precisely, even after long-term storage when kept at −80°C.

We then used the adhesive sheet for bacterial monitoring in Kibo. Samples were collected as part of the “Microbe” research program. We determined bacterial numbers with different approaches of fluorescence microscopy and quantitative PCR targeting the 16S rRNA gene to confirm the reliability of the results. Table 2 shows the bacterial abundance in Kibo determined with these two methods. Before this experiment, we confirmed that the number of 16S rRNA genes on an unused adhesive sheet (negative control) was below the quantification limit. Using fluorescent staining, bacterial abundance at each site was equivalent to or less than the quantification limit. Using quantitative PCR, a similar result to fluorescent staining was obtained, except on the return air grill. The return air grill sample collected a lot of dust on the adhesive face; therefore, we were not able to discriminate bacterial cells from other particles clearly under an epifluorescent microscope. Quantitative PCR was more effective when the adhesive face was covered with dust.

**Fig. 4:**
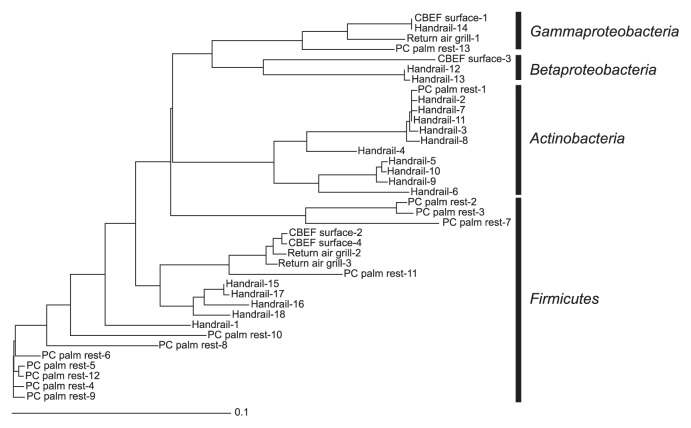
Neighbor-joining tree of the 16S rRNA gene sequences retrieved from bacteria collected in Kibo, Japanese Experiment Module on the ISS.

Finally, we estimated the taxonomic distribution of bacteria collected from surfaces at four sites in Kibo. The yield of PCR products with a 8f/1492r primer set or an EUB f933-GC-clamp/EUB r1387 primer set was too low to construct 16S rRNA gene clone libraries or perform PCR-DGGE followed by sequencing, respectively; therefore, we used nested PCR-DGGE followed by sequencing to obtain phylogenetic information of bacteria present in Kibo. This approach showed the presence of bacteria, although it did not reflect the dominance of any particular species (15). Fig. 4 shows the phylogenetic tree of the 16S rRNA gene fragments retrieved from a DGGE gel. Their phylogenetic affiliations determined by a BLAST search are also shown in Table S1. Bacteria in the phyla *Actinobacteria* and *Firmicutes* were frequently detected on the surface of the PC palm rest and handrail, which astronauts touch frequently. Most of these bacteria have been detected on the hands as human skin microbiome (5); thus, bacterial cells might transfer to the surfaces in Kibo via astronaut contact. Furthermore, this result was consistent with previous research in other modules of the ISS (13). Other bacteria detected in Kibo, such as *Beta*- and *Gammaproteobacteria*, are also part of human skin microflora (5).

The abundance of bacteria in Kibo, except on the return air grill, was equivalent to or lower than that on the surfaces in our laboratory (10^5^ cells [cm^2^]^−1^) (20), and bacteria detected in Kibo was related to the human skin microflora. Furthermore, with regard to fungal biota analyses, Satoh *et al.* (16) reported that the degree of cleanliness in Kibo during the first 460 d was equivalent to that in a clean room environment on the ground. The surfaces of equipment installed in Kibo are wiped with disinfectant once a week; however, on the return air grill it is easy for dust to accumulate, so the number of microbes can be higher on its surface. From these aspects of microbial abundance and their phylogenetic affiliation, Kibo has been microbiologically well maintained during the first 880 d; however, microbial abundance in Kibo may increase with the prolonged stay of astronauts. To ensure crew safety and understand bacterial dynamics in space habitation environments, continuous bacterial monitoring in Kibo is required.

## Supplementary Material



## Figures and Tables

**Table 1:** Primers and probes used in this study

Primer	Target gene and position	Sequence (5′ to 3′)	References
8f	16S rRNA, 8–27^c^	AGAGTTTGATCCTGGCTCAG	(9)
1492r	16S rRNA, 1510–1492^c^	GGTTACCTTGTTACGACTT	(9)
EUB f933	16S rRNA, 933–954^c^	GCACAAGCGGTGGAGCATGTGG	(6)
EUB f933-GC-clamp	16S rRNA, 933–954^c^	CGCCCGCCGCGCGCGGCGGGCGGGGCGGG GGCACGGGGGG-EUB f933	(6)
EUB r1387	16S rRNA, 1387–1368^c^	GCCCGGGAACGTATTCACCG	(6)
pgL1908f	*luc* gene, 1908–1927^d^	AGGAAGCTTTCCATGGAAGA	(12)
Luc175r	*luc* gene, 2063–2082^d^	CAGCGTAAGTGATGTCCACC	(12)
n-LucHP1^a^	*luc* gene, 2008–2029^d^	TGAAGAGATACGCCCTGGTTCC	(12)
n-LucHP2^b^	*luc* gene, 2030–2058^d^	GGAACAATTGCTTTTACAGATGCACATA	(12)

a 3′ FITC labeled;

b 5′ Light Cycler Red 640 labeled;

c *Escherichia coli* numbering system;

d Numbering bases of pGeneGRIP-Luc.

**Table 2:** Bacterial abundance on the interior surfaces in Kibo determined with fluorescent staining and quantitative PCR

Sampling point	Bacterial abundance (cells [cm^2^]^−1^)	

	Fluorescent staining	Quantitative PCR^a^,^b^
CBEF surface	<2.0×10^4^	<7×10^4^ (<7×10^4^)
PC palm rest	2.6×10^4^	3×10^3^–5×10^4^ (5×10^4^)
Return air grill	Not countable	5×10^6^–8×10^7^ (8×10^7^)
Handrail	2.8×10^4^	2×10^3^–3×10^4^ (3×10^4^)

a Bacterial cells carry 1 to 15 copies of the 16S rRNA gene in their genome (8).

b Numbers in parentheses show 16S gene copy numbers (unit: copies [cm^2^]^−1^).
