# Supplementary material for: Bacterial Monitoring with Adhesive Sheet in the International Space Station-“Kibo”, the Japanese Experiment Module
Source: Microbes Environ. 2013 Apr 20;28(2):264–8. doi: 10.1264/jsme2.ME12184 (PMC4070660; doi:10.1264/jsme2.ME12184)
Supplement: Supplementary file 1 [file 28_264_s1.pdf]

Table S1. Phylogenetic affiliation of DGGE bands originating from bacteria collected from the interior surfaces in the Kibo

Sample (Accesion No.)	Most closely related species (Accession No.)	Similarity (%)	Phylogenetic affiliation				
			Phylum	Class	Order	Family	Genus
CBEF_surface-1 (AB720834)	*Escherichia fergusonii* (NR_027549)	100	*Proteobacteria*	*Gammaproteobacteria*	*Enterobacteriales*	*Enterobacteriaceae*	*Escherichia/Shigella*
	Escherichia albertii (NR_025569)	100	*Proteobacteria*	*Gammaproteobacteria*	*Enterobacteriales*	*Enterobacteriaceae*	*Escherichia/Shigella*
	Escherichia coli (NR_024570)	100	*Proteobacteria*	*Gammaproteobacteria*	*Enterobacteriales*	*Enterobacteriaceae*	*Escherichia/Shigella*
CBEF_surface-2 (AB720835)	*Staphylococcus warneri* (NR_025922)	99	*Firmicutes*	*Bacilli*	*Bacillales*	*Staphylococcaceae*	*Staphylococcus*
	Staphylococcus pasteuri (NR_024669)	99	*Firmicutes*	*Bacilli*	*Bacillales*	*Staphylococcaceae*	*Staphylococcus*
CBEF_surface-3 (AB720836)	*Ralstonia solanacearum* (NR_044040)	96	*Proteobacteria*	*Betaproteobacteria*	*Burkholderiales*	*Burkholderiaceae*	*Ralstonia*
	Ralstonia pickettii (NR_043152)	96	*Proteobacteria*	*Betaproteobacteria*	*Burkholderiales*	*Burkholderiaceae*	*Ralstonia*
	Ralstonia insidiosa (NR_025242)	96	*Proteobacteria*	*Betaproteobacteria*	*Burkholderiales*	*Burkholderiaceae*	*Ralstonia*
	Ralstonia syzygii (NR_040803)	96	*Proteobacteria*	*Betaproteobacteria*	*Burkholderiales*	*Burkholderiaceae*	*Ralstonia*
CBEF_surface-4 (AB720837)	*Staphylococcus warneri* (NR_025922)	99	*Firmicutes*	*Bacilli*	*Bacillales*	*Staphylococcaceae*	*Staphylococcus*
	Staphylococcus pasteuri (NR_024669)	99	*Firmicutes*	*Bacilli*	*Bacillales*	*Staphylococcaceae*	*Staphylococcus*
PC_palm_rest-1 (AB720838)	*Propionibacterium acnes* (NR_040847)	99	*Actinobacteria*	*Actinobacteria*	*Actinomycetales*	*Propionibacteriaceae*	*Propionibacterium*
PC_palm_rest-2 (AB720839)	*Veillonella parvula* (NR_043332)	99	*Firmicutes*	*Negativicutes*	*Selenomonadales*	*Veillonellaceae*	*Veillonella*
PC_palm_rest-3 (AB720840)	*Veillonella parvula* (NR_043332)	99	*Firmicutes*	*Negativicutes*	*Selenomonadales*	*Veillonellaceae*	*Veillonella*
PC_palm_rest-4 (AB720841)	*Streptococcus oralis* (NR_042927)	99	*Firmicutes*	*Bacilli*	*Lactobacillales*	*Streptococcaceae*	*Streptococcus*
	Streptococcus pneumoniae (NR_028665)	99	*Firmicutes*	*Bacilli*	*Lactobacillales*	*Streptococcaceae*	*Streptococcus*
PC_palm_rest-5 (AB720842)	*Streptococcus pseudopneumoniae* (NR_027214)	99	*Firmicutes*	*Bacilli*	*Lactobacillales*	*Streptococcaceae*	*Streptococcus*
PC_palm_rest-6 (AB720843)	*Streptococcus sanguinis* (NR_024841)	99	*Firmicutes*	*Bacilli*	*Lactobacillales*	*Streptococcaceae*	*Streptococcus*
PC_palm_rest-7 (AB720844)	*Megasphaera micronuciformis* (NR_025230)	99	*Firmicutes*	*Negativicutes*	*Selenomonadales*	*Veillonellaceae*	*Megasphaera*
PC_palm_rest-8 (AB720845)	*Streptococcus constellatus* (NR_042833)	93	*Firmicutes*	*Bacilli*	*Lactobacillales*	*Streptococcaceae*	*Streptococcus*
	Streptococcus constellatus (NR_041721)	93	*Firmicutes*	*Bacilli*	*Lactobacillales*	*Streptococcaceae*	*Streptococcus*
PC_palm_rest-9 (AB720846)	*Streptococcus australis* (NR_036936)	99	*Firmicutes*	*Bacilli*	*Lactobacillales*	*Streptococcaceae*	*Streptococcus*
	Streptococcus oralis (NR_042927)	99	*Firmicutes*	*Bacilli*	*Lactobacillales*	*Streptococcaceae*	*Streptococcus*
	Streptococcus pneumoniae (NR_028665)	99	*Firmicutes*	*Bacilli*	*Lactobacillales*	*Streptococcaceae*	*Streptococcus*
PC_palm_rest-10 (AB720847)	*Streptococcus phocae* (NR_042227)	91	*Firmicutes*	*Bacilli*	*Lactobacillales*	*Streptococcaceae*	*Streptococcus*
	Streptococcus pseudoporcinus (NR_043704)	91	*Firmicutes*	*Bacilli*	*Lactobacillales*	*Streptococcaceae*	*Streptococcus*
	Streptococcus pseudopneumoniae (NR_027214)	91	*Firmicutes*	*Bacilli*	*Lactobacillales*	*Streptococcaceae*	*Streptococcus*
	Streptococcus cristatus (NR_042771)	91	*Firmicutes*	*Bacilli*	*Lactobacillales*	*Streptococcaceae*	*Streptococcus*
	Streptococcus agalactiae (NR_040821)	91	*Firmicutes*	*Bacilli*	*Lactobacillales*	*Streptococcaceae*	*Streptococcus*
PC_palm_rest-11 (AB720848)	*Staphylococcus capitis* (NR_027519)	93	*Firmicutes*	*Bacilli*	*Bacillales*	*Staphylococcaceae*	*Staphylococcus*
	Staphylococcus capitis (NR_036775)	93	*Firmicutes*	*Bacilli*	*Bacillales*	*Staphylococcaceae*	*Staphylococcus*
PC_palm_rest-12 (AB720849)	*Streptococcus pseudopneumoniae* (NR_027214)	99	*Firmicutes*	*Bacilli*	*Lactobacillales*	*Streptococcaceae*	*Streptococcus*
PC_palm_rest-13 (AB720850)	*Aggregatibacter aphrophilus* (NR_042876)	98	*Proteobacteria*	*Gammaproteobacteria*	*Pasteurellales*	*Pasteurellaceae*	*Aggregatibacter*
return_grill-1 (AB720851)	*Erwinia pyrifoliae* (NR_044101)	97	*Proteobacteria*	*Gammaproteobacteria*	*Enterobacteriales*	*Enterobacteriaceae*	*Erwinia*
return_grill-2 (AB720852)	*Staphylococcus simiae* (NR_043146)	100	*Firmicutes*	*Bacilli*	*Bacillales*	*Staphylococcaceae*	*Staphylococcus*
return_grill-3 (AB720853)	*Staphylococcus caprae* (NR_024665)	100	*Firmicutes*	*Bacilli*	*Bacillales*	*Staphylococcaceae*	*Staphylococcus*
handrail-1 (AB720854)	*Granulicatella adiacens* (NR_025862)	99	*Firmicutes*	*Bacilli*	*Lactobacillales*	*Carnobacteriaceae*	*Granulicatella*
	Granulicatella elegans (NR_028682)	99	*Firmicutes*	*Bacilli*	*Lactobacillales*	*Carnobacteriaceae*	*Granulicatella*
handrail-2 (AB720855)	*Propionibacterium acnes* (NR_040847)	99	*Actinobacteria*	*Actinobacteria*	*Actinomycetales*	*Propionibacteriaceae*	*Propionibacterium*
handrail-3 (AB720856)	*Propionibacterium acnes* (NR_040847)	99	*Actinobacteria*	*Actinobacteria*	*Actinomycetales*	*Propionibacteriaceae*	*Propionibacterium*
handrail-4 (AB720857)	*Propionibacterium acnes* (NR_040847)	93	*Actinobacteria*	*Actinobacteria*	*Actinomycetales*	*Propionibacteriaceae*	*Propionibacterium*
handrail-5 (AB720858)	*Rothia mucilaginosa* (NR_044873)	99	*Actinobacteria*	*Actinobacteria*	*Actinomycetales*	*Micrococcaceae*	*Rothia*
handrail-6 (AB720859)	*Tsukamurella inchonensis* (NR_041804)	96	*Actinobacteria*	*Actinobacteria*	*Actinomycetales*	*Tsukamurellaceae*	*Tsukamurella*
	Tsukamurella spumae (NR_044951)	96	*Actinobacteria*	*Actinobacteria*	*Actinomycetales*	*Tsukamurellaceae*	*Tsukamurella*
handrail-7 (AB720860)	*Propionibacterium acnes* (NR_040847)	99	*Actinobacteria*	*Actinobacteria*	*Actinomycetales*	*Propionibacteriaceae*	*Propionibacterium*
handrail-8 (AB720861)	*Propionibacterium acnes* (NR_040847)	99	*Actinobacteria*	*Actinobacteria*	*Actinomycetales*	*Propionibacteriaceae*	*Propionibacterium*
handrail-9 (AB720862)	*Rothia mucilaginosa* (NR_044873)	99	*Actinobacteria*	*Actinobacteria*	*Actinomycetales*	*Micrococcaceae*	*Rothia*
handrail-10 (AB720863)	*Rothia mucilaginosa* (NR_044873)	100	*Actinobacteria*	*Actinobacteria*	*Actinomycetales*	*Micrococcaceae*	*Rothia*
handrail-11 (AB720864)	*Propionibacterium acnes* (NR_040847)	100	*Actinobacteria*	*Actinobacteria*	*Actinomycetales*	*Propionibacteriaceae*	*Propionibacterium*
handrail-12 (AB720865)	*Neisseria subflava* (NR_041989)	99	*Proteobacteria*	*Betaproteobacteria*	*Neisseriales*	*Neisseriaceae*	*Neisseria*
handrail-13 (AB720866)	*Neisseria subflava* (NR_041989)	99	*Proteobacteria*	*Betaproteobacteria*	*Neisseriales*	*Neisseriaceae*	*Neisseria*
handrail-14 (AB720867)	*Escherichia fergusonii* (NR_027549)	100	*Proteobacteria*	*Gammaproteobacteria*	*Enterobacteriales*	*Enterobacteriaceae*	*Escherichia/Shigella*
	Escherichia albertii (NR_025569)	100	*Proteobacteria*	*Gammaproteobacteria*	*Enterobacteriales*	*Enterobacteriaceae*	*Escherichia/Shigella*
	Escherichia coli (NR_024570)	100	*Proteobacteria*	*Gammaproteobacteria*	*Enterobacteriales*	*Enterobacteriaceae*	*Escherichia/Shigella*
handrail-15 (AB720868)	*Bacillus nealsonii* (NR_044546)	99	*Firmicutes*	*Bacilli*	*Bacillales*	*Bacillaceae*	*Bacillus*
handrail-16 (AB720869)	*Bacillus nealsonii* (NR_044546)	95	*Firmicutes*	*Bacilli*	*Bacillales*	*Bacillaceae*	*Bacillus*
	Bacillus soli (NR_025591)	95	*Firmicutes*	*Bacilli*	*Bacillales*	*Bacillaceae*	*Bacillus*
	Bacillus bataviensis (NR_036766)	95	*Firmicutes*	*Bacilli*	*Bacillales*	*Bacillaceae*	*Bacillus*
handrail-17 (AB720870)	*Bacillus nealsonii* (NR_044546)	98	*Firmicutes*	*Bacilli*	*Bacillales*	*Bacillaceae*	*Bacillus*
handrail-18 (AB720871)	*Bacillus pseudalcaliphilus* (NR_026145)	98	*Firmicutes*	*Bacilli*	*Bacillales*	*Bacillaceae*	*Bacillus*
